# Characteristics of in Vivo Model Systems for Ovarian Cancer Studies

**DOI:** 10.3390/diagnostics9030120

**Published:** 2019-09-14

**Authors:** Patrycja Tudrej, Katarzyna Aleksandra Kujawa, Alexander Jorge Cortez, Katarzyna Marta Lisowska

**Affiliations:** Center for Translational Research and Molecular Biology of Cancer, Maria Skłodowska-Curie Institute—Oncology Center, Gliwice Branch, , ul. Wybrzeże Armii Krajowej 15, 44-101 Gliwice, Poland; patrycja.tudrej@io.gliwice.pl (P.T.); katarzyna.kujawa@io.gliwice.pl (K.A.K.); alexander.cortez@io.gliwice.pl (A.J.C.)

**Keywords:** ovarian cancer, in vivo model systems, *Drosophila melanogaster*, South African clawed frog (*Xenopus laevis*, *Xenopus tropicalis*), syngeneic mice model, patient derived xenograft (PDX) model, genetically engineered mice, laying hen model, chic embryo chorioallantoic membrane (CAM) model

## Abstract

An understanding of the molecular pathogenesis and heterogeneity of ovarian cancer holds promise for the development of early detection strategies and novel, efficient therapies. In this review, we discuss the advantages and limitations of animal models available for basic and preclinical studies. The fruit fly model is suitable mainly for basic research on cellular migration, invasiveness, adhesion, and the epithelial-to-mesenchymal transition. Higher-animal models allow to recapitulate the architecture and microenvironment of the tumor. We discuss a syngeneic mice model and the patient derived xenograft model (PDX), both useful for preclinical studies. Conditional knock-in and knock-out methodology allows to manipulate selected genes at a given time and in a certain tissue. Such models have built our knowledge about tumor-initiating genetic events and cell-of-origin of ovarian cancers; it has been shown that high-grade serous ovarian cancer may be initiated in both the ovarian surface and tubal epithelium. It is postulated that clawed frog models could be developed, enabling studies on tumor immunity and anticancer immune response. In laying hen, ovarian cancer develops spontaneously, which provides the opportunity to study the genetic, biochemical, and environmental risk factors, as well as tumor initiation, progression, and histological origin; this model can also be used for drug testing. The chick embryo chorioallantoic membrane is another attractive model and allows the study of drug response.

## 1. Introduction

Advances in the diagnosis and treatment of ovarian cancer require a better understanding of this disease. A major finding of recent years is the recognition that different histological types of ovarian cancer can originate from distinct epithelia and, in fact, represent distinct disease entities (reviewed in the works of [1,2,3]). However, the question about the tissue/cell of origin of each histological type has not been definitely resolved. Some very interesting results were achieved using animal models, in particular, genetically modified mice. Domestic hen is also suitable for these investigations.

The results of ovarian cancer treatment have improved only slightly within the last decades. An obvious milestone was the introduction of platinum-taxanes standard chemotherapy. Further gradual improvement has been achieved owing to the development of better technical opportunities and improved surgical skills, resulting in increased numbers of completely debulked patients and better survival [4]. The newest milestone in ovarian cancer treatment is inhibitors of polyADP-ribose polymerase (PARP), which belong to the newer class of drugs, called biological drugs. Their mechanism of action is exploiting the synthetic lethality phenomenon. It was shown in clinical trials that drugs such as Olaparib or Niraparib extend progression-free survival for several months and even years (reviewed in the work of [5]).

The success of PARPi encourages further investigations for new biological drugs. Many studies are ongoing on novel drugs designed against different molecular targets in ovarian cancer. New molecular targets are sought mainly among the proteins that participate in signaling pathways engaged in regulation of crucial cellular processes, such as proliferation, migration, adhesion, differentiation, cell death, and so on. *Drosophila melanogaster* is widely used as a convenient model for studying cellular signaling pathways. In the context of ovarian cancer, the most intensively studied are the so-called border cells from *Drosophila* ovary.

Different experimental immunotherapy approaches are also widely tested, including passive or active immunotherapy, enhancement of unspecific immune response, and immune checkpoint inhibitors (reviewed in the work of [5]). It is suggested that South African clawed frog (*Xenopus)* models could be adapted for studies on tumor immunity and anticancer immune response [6].

Before new inhibitors/targeted drugs can enter clinical trials, they should be tested in the preclinical setting. Animal models suitable for this purpose comprise the syngeneic mouse model as well as cancer xenografts in immunocompromised mice, including patient-derived xenografts (PDX). Laying hen can also be used for drug testing, as well as for the studies on risk factors and prevention strategies [7]. Another model, very useful for studies on efficacy of new therapeutic approaches, is chicken egg chorioallantoic membrane [8].

In this review, we will discuss these models and their suitability for investigation of particular aspects of ovarian cancer. We will also mention the results of the most important studies done with the aid of these models.

## 2. Animal Models for Ovarian Cancer Studies

In ovarian cancer studies, three species are most commonly used: fruit fly, mouse, and lying hen. There is no single, universal model that perfectly recapitulates every stage of this disease in humans. Some models are suitable for studies on cellular signaling and tumor initiation, while others allow to investigate the mechanisms of peritoneal metastases and ascites formation, and so on. Each model has its own constraints; when designing the experiment, we should bear in mind the pros and cons of each one and choose that most suitable for a particular type of investigation (Table 1).

### 2.1. Fruit Fly Model

Fruit fly (*Drosophila melanogaster*) has short life cycle and is easy to propagate, thus it is a convenient and cheap model organism. Although there are fundamental anatomical and physiological differences between mammals and *Drosophila*, the mechanisms regulating molecular signaling pathways are highly evolutionarily conserved and demonstrate significant similarity. Thus, fruit fly can serve as a model to study molecular mechanisms involved in ovarian cancer initiation and progression (reviewed in the works of [10,11]).

**Figure 1:**
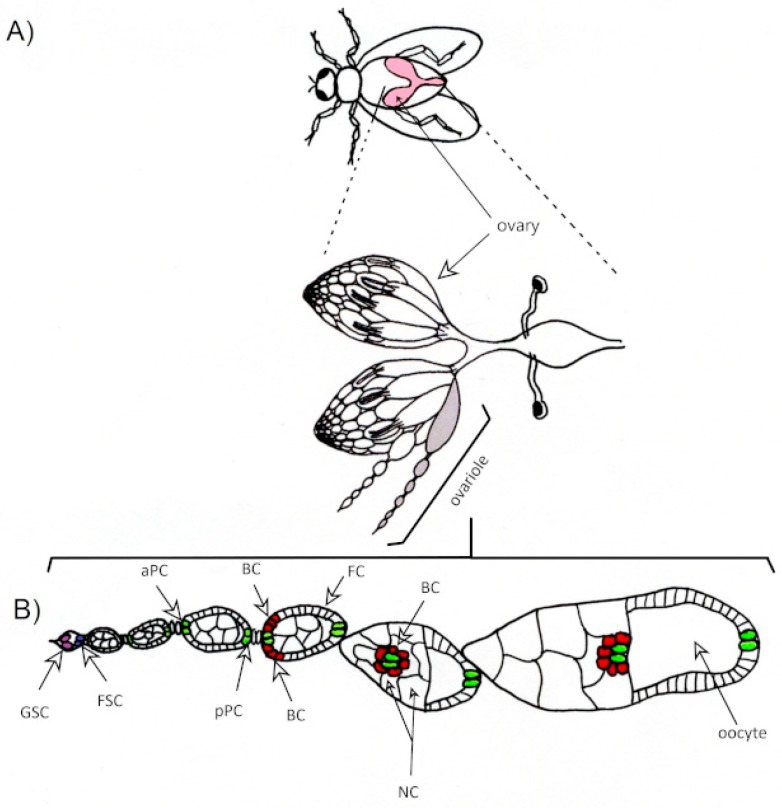
(**A**) Schematic diagram of reproductive system of *Drosophila melanogaster.* (**B**) Migration of border cells in the developing ovarian follicle. GSC—germline stem cell, FSC—follicle stem cell, aPC—anterior polar cell, pPC—posterior polar cell, BC—border cell, FC—follicle cell, NC—nurse cell.

The *Drosophila* has two ovaries, each composed of 6−18 ovarian tubules (ovarioles). The so-called germarium is localized in the apical end of each ovariole. It contains two to four germline stem cells (GSCs) and two follicle stem cells (FSCs). During oogenesis, the egg chamber is gradually formed. It contains 16 germline cells, 1 of which will convert into the oocyte, while the 15 others become nurse cells. These germline cells are surrounded by the monolayer of follicular epithelium, which is derived from somatic FSCs. The follicular epithelium is thought to be a counterpart of the human ovarian surface epithelium (OSE). Two follicular cells at each pole of egg chamber convert themselves into specialized pole cells; they stop dividing and start paracrine signaling. Apical pole cells recruit several (4−8) adjacent follicular cells, referred to as border cells (BCs). Together, they form the syncytium and coordinately migrate toward the oocyte, where they place themselves on its surface and form micropyle, the structure that is required for sperm entry into the oocyte during fertilization (Figure 1).

The transformation of follicular cells into border cells requires epithelial-to-mesenchymal transition (EMT). When BCs migrate toward oocyte, they must demonstrate invasive behavior, in order to invade and pass between nurse cells. They also have to remodel their cell-to-cell junctions and their adhesive properties when they detach from follicular epithelium. All these processes are analogous to those taking place during ovarian cancer metastasis in higher animals. Thus, fruit fly border cells are recognized as a model suitable to study these mechanisms. Another analogy concerns the fact that BCs migrate as clusters, similar to ovarian cancer cells that form aggregates when floating in the malignant ascites.

It was shown that BCs’ recruitment, EMT, and migration are regulated by Jak/Stat signaling cascade [12,13,14,15,16]. First, apical pole cells secrete the Upd (Unpaired) cytokine, the main ligand of Domeless receptor. Domeless is the only receptor in the Jak/Stat pathway in *Drosophila*. Jak/Stat pathway activation is achieved in neighboring follicular cells. Then, the expression of several downstream genes is activated, among them is that of the transcription factor C/EBP, which is indispensable for the initiation of BCs’ migration. In humans, one of the STAT family proteins, STAT3, is found constitutively phosphorylated in several tumors and tumor cell lines, among them in ovarian cancer lines. Some genes regulated by Stat3 are engaged in metastatic process; it was shown using the siRNA technique that STAT3 inhibition or depletion results in a diminished number of metastases (reviewed in the works of [10,11]).

Taiman is another protein involved in the control of BCs’ migration. Its counterpart in humans is AIB1/NCOA3 (amplified in breast 1/nuclear receptor coactivator 3), co-activator of the estrogen receptor (ER) [17]. *AIB1* gene amplification is found in a large percentage of human breast and ovarian cancers [18]. In *Drosophila*, Taiman protein initiates migration of BCs in response to rising levels of ecdysone, a steroid hormone, by co-activating its receptor [19]. In mice, overexpression of *Aib1* leads to dysplasia of mammary glands and development of mammary cancers [20]. In humans, *AIB1* overexpression in ovarian cancer was shown to be associated with shorter survival [21].

As steroid hormones are involved in the regulation of BCs’ migration, the question remains about their role in ovarian cancer. The role of steroid hormones in the development of human reproductive tract cancers is ambiguous. On the basis of epidemiological studies, it was suggested that estrogen-based single-component hormone replacement therapy (HRT) is related with an increased risk of ovarian cancer. Despite conflicting results, it seems that most postmenopausal women presenting high levels of 17β-estradiol, testosterone, and estrone are at potential risk of developing ovarian, breast, and endometrial cancers (reviewed in the work of [22]). Moreover, a recent study [23] by Collaborative Group on Epidemiological Studies of Ovarian Cancer (CGESOC), which analyzed 52 trials performed between 1977 and 2013, has shown slightly increased ovarian cancer risk, despite the HRT type (single- or two-component, with estrogen and progesterone) and duration (even when HRT was shorter than five years). Interestingly, an increased risk was observed for serous and endometrioid cancers, while for clear-cell and mucinous histological types, a protective effect was observed. Endocrine therapy is investigated for the treatment of serous ovarian cancers, both LGSOC and HGSOC, which have the highest percentage of ER-positive tumors among all histological types (reviewed in the works of [24,25]). It is suggested that endocrine therapy could be considered as a viable strategy to defer subsequent chemotherapy for relapsed HGSOC. Patients with high ER expression in the tumor and a treatment free interval ≥180 days are most likely to benefit [26]. Also, HGSOC patients with platinum resistant/refractory tumors are considered eligible for endocrine therapy. Preliminary data suggest that LGSOC patients may benefit from endocrine therapy [27].

Fruit fly was also used in the studies on *Yap* (Yes-associated protein) oncogene. In humans, *YAP* is engaged in promoting ovarian carcinogenesis by regulating the Hippo pathway. In *Drosophila*, this pathway regulates follicular cell polarization and cytoskeleton dynamics in border cells during their migration [28,29]. Moreover, it was found that the Hippo pathway plays a crucial role in the control of cell proliferation and differentiation of both in *Drosophila* and mammals (reviewed in the works of [30,31]). In humans, high nuclear expression of YAP in ovarian cancer cells, together with low cytoplasmic level of phosphorylated YAP, was shown to be an independent negative prognostic factor and was associated with significantly shorter patient survival [32]. It was shown that overexpression of human *YAP* induced tumorigenesis in the *Drosophila* ovary. Functional in vitro studies revealed that *YAP* overexpression in OSE cells stimulates their proliferation, migration rate, anchorage-independent growth, and apoptosis-resistance under cisplatin treatment. On the contrary, *YAP* silencing results in the increased susceptibility to apoptosis in response to cisplatin. The authors of these experiments postulate that *YAP* is an oncogene responsible for ovarian cancer progression and development of chemoresistance [32,33].

Border cells express two receptor tyrosine kinases: epidermal growth factor receptor (Egfr) and Pvr, which shows similarity to the human platelet-derived growth factor receptor (PDGFR) and Vascular endothelial growth factor receptor (VEGFR). These receptors bind several ligands (PVF1, Spitz, Keren and Gurken) that are secreted by germ cells, in particular by the ovary. Activation of *Egfr* and *Pvr* by these ligands is required to guide border cells to their destination, thus indicating the role of these receptors in the invasion process (reviewed in the work of [34]).

Overexpression of EGFR is observed in the majority of ovarian cancers; its signaling increases cellular motility, invasiveness, and proliferation of ovarian cancer cells. An increased level of vascular endothelial growth factor (VEGF) is related to tumor progression, resulting from enhanced angiogenesis and emerging vasculature of the tumor. VEGF is also responsible for the production of ascites, owing to increased vascular permeability. Recent findings confirm that EGFR signaling enhances the migration of human ovarian cancer cells, while VEGF can also play a role in tumor progression via stimulation of proliferation and migration rate of cancer cells. As it was shown that VEGF activates the STAT pathway in aortic endothelial cells, it is also postulated that VEGF could activate STATs in tumor cells [35]. Thus, it is possible that anti-VEGF therapy, besides its anti-angiogenic activity, can also exert a direct antiproliferative effect (reviewed in the work of [11]). Double anti-tyrosine kinase inhibitor AEE788, which is able to block EGFR and VEGFR simultaneously, was already tested in the orthotropic mouse model of ovarian cancer. Preliminary results indicate that combination of AEE788 with taxanes or docetaxel is highly effective [36,37].

Studies in *Drosophila* showed that protein homologous to the human BRCA2 is indispensable for the accurate homologous recombination during mitosis and meiosis, as well as for appropriate activation of cell cycle checkpoints in meiosis [38]. Some genes, for example, *Psc* (Posterior sex combs) and *Su(z)2* (Suppressor of zeste two) from the Polycomb group, which control the proliferation of germ and follicular cells in *Drosophila*, were further identified as potential oncogenes/suppressor genes in humans. Their mechanisms of action were elucidated using the fruit fly model [11,39]. Human orthologs of *Psc* and *Su(z)2* are *BMI1* (BMI1 Proto-Oncogene) and *PCGF2* (Polycomb Group Ring Finger 2), respectively.

### 2.2. Mouse Models

Mice (*Mus musculus*) are the most widely used animals for human disease modelling. The advantage of murine models results from the similarity of its physiology and molecular signaling pathways to those of humans, as well as from the large collection of mouse strains dedicated to studies of different diseases and molecular mechanisms. Other pros are high fertility, short life-span, quick development, and low maintenance requirements (except for immunocompromised mice). The most obvious difficulty in the development of a stable model of ovarian cancer arises from the fact that the disease results in infertility.

Mice do not develop ovarian cancer spontaneously, thus using a mice model requires tumor induction by grafting. For induction of ovarian cancer, several compounds were used, for example, 7,12-dimethylbenz(a)anthracene (DMBA), 20-methylcholanthrene, 1,3-butadiene, formic acid 2-[4-(5-nitro-2-furyl)-2-thiazolyl]hydrazide, and N-methyl-N’-nitrosourea; however, their efficiency was low. At present, the models with tumors induced chemically or hormonally are rarely used, because their histopathological features are difficult to predict, similarly to their molecular profiles.

The most frequently used are mice bearing xenotransplanted human tumors, syngeneic models, and genetically modified animals (reviewed in the works of [40,41]). Xenotransplanted and syngeneic tumors enable the study of early disease stages, as well as invasion and spreading of cancer cells. On the contrary, genetically modified mice are particularly well suited for studies on mechanisms of tumor initiation.

#### 2.2.1. Xenograft Models

The first attempts to xenotransplant human cancer cells to the immunocompromised mice started in the early 1960s. Mice with bone marrow damaged by ionizing radiation were used in these early experiments. At present, numerous mice strains are available with decreased immunological response. Some of them originate from animals with naturally occurring genetic disorders, like nude mice (Foxn1 Nu/Nu, with spontaneous deletion in forkhead box N1 gene), which have low levels of T lymphocytes; SCID (severe combined immunodeficiency) mice, depleted of functional B and T lymphocytes; or NOD/SCID (non-obese diabetic/severe combined immunodeficiency). NOD/SCID IL2Rγ^null^ (NSG) mice have additionally impaired cytokine signaling, which affects natural killer (NK) cells’ differentiation (reviewed in the work of [42]). These strains achieved great popularity, as they are convenient hosts for cancer transplants.

Xenotransplants are appropriate for fast-growing tumors. These models are widely used for the analysis of cancer cells’ tumorigenicity, tumor histology, and for testing of tumor response to novel therapies. For xenotransplantations, either established or primary human ovarian cancer cell lines can be used, as well as ex vivo transplants. Cancer cells or tumor explants can be engrafted subcutaneously, intraperitoneally, or orthotopically, that is, to the anatomical location from which they were originally derived (Table 2). Some of these models are suitable for the studies on early stages of cancer, while others allow the study of invasion and metastases (reviewed in the works of [42,43]).

After subcutaneous xenograft, the tumor formation is confined to the place of injection. The tumor is formed within several weeks and usually shows histology similar to the original tumor. Its emergence can be recognized by eye, its size can also be easily measured; thus, it is a comfortable model for studies of drug-response. The shortcoming of the subcutaneous model is concerned with the fact that the tumor does not develop in the right anatomic location and microenvironment.

Unlike the human ovary, its mouse counterpart is encapsulated in the membranous structure called *bursa ovari*. In the orthotopic model, cancer cells or explants can be delivered into this space. The initial phases of tumor development are confined to the bursa; later, metastases can occur and ascites formation may be observed. This model is, however, technically challenging (reviewed in the work of [44]); leakage of cancer cells may occur after intra-bursal injection, making it difficult to distinguish between true metastases and tumors arising from accidentally splashed cancer cells. Owing to the anatomic differences between mice and humans, who do not have ovarian bursa, this model is also not adequate for the investigation of metastatic process.

Intraperitoneal injections allow obtaining a disseminated cancer model; cancer foci are quickly formed within the peritoneum, on the liver and spleen surface, similar to the advanced stages of human ovarian cancer. In orthotropic and intraperitoneal models, the monitoring of tumor growth is complicated. It requires advanced imaging methods, while transportation of immunocompromised mice to the imaging unit outside from the controlled environment may increase the risk of infection.

##### Xenografts Using Established Cell Lines

Established cell lines should be precisely chosen for xenotransplantation experiments. Ovarian cancer heterogeneity, and its consequences, must be taken into account (reviewed in the works of [45,46]). Many ovarian cancer cell lines were established dozens of years ago, when the knowledge about substantial etiological, molecular, and clinical differences between distinct histological types was lacking. Thus, some of very common cell lines were not described precisely according to their histological origin. In recent years, attempts were undertaken to verify backwards the origin of these cell lines, based on their molecular profile. However, there are still many discrepancies. Most importantly, among the most frequently cited cell lines, there is no reliable model of the high-grade serous ovarian cancer (HGSOC). The most popular cell line, SKOV3, is most probably derived from clear cell ovarian cancer; the second most prevalent, A2780, is derived from endometrioid cancer.

Two rather unknown cell lines KURAMOCHI and OVSAHO are suggested as the most reliable models for HGSOC. Among other lines, OVCAR4 is indicated as HGSOC [47] and, with some doubt, also OVCAR3 [48]. The OVPA8 cell line established by our team is also derived from a patient with histologically confirmed HGSOC, and our detailed in vitro characteristics of these cells also supports HGSOC origin [49]; this line will be soon available from European Collection of Authenticated Cell Cultures (ECACC; accession No. 19061601 and 19061602).

The discrepancies concerning cellular/histological origin of several ovarian cancer cell lines are reflected, for example, by the fact that some cells inoculated into mice result in tumors with histology distinct from that ascribed to them. For example, the HEY line, derived from moderately differentiated papillary cyst, results in undifferentiated tumors in nude mice [50]. The A2780 cell line, derived from endometrioid adenocarcinoma [51], gives rise to undifferentiated carcinomas [52]. The SKOV3 line, in many studies wrongly labeled as serous, gives clear-cell tumors [50]. Only OVCAR3 cells, which are described as derived from poorly differentiated papillary adenocarcinoma [51], give such tumors in nude mice [52].

Xenotransplant model obtained with OVCAR3 cells was used for preclinical testing of antiangiogenic therapy. It was shown that monoclonal antibody against human VEGF is effective in suppressing ascites’ formation. Combination of paclitaxel and VEGF inhibitors showed a synergistic effect, resulting in suppression of tumor growth and reduction of ascites. These observations were later confirmed in clinical trials that tested bevacizumab in monotherapy and in combination with paclitaxel [5,53].

Interestingly, cells from different cell lines have distinct preferences according to the place of injection. Hernandez et al., [54] tested 18 established ovarian cancer cell lines in this respect. They showed that SKOV3 cells are highly tumorigenic after subcutaneous and intraperitoneal injection, while OVCAR3 and OVCAR4 have low subcutaneous tumorigenicity and intermediate intraperitoneal tumorigenicity, and do not form tumors when injected intrabursally. A2780 cells have intermediate subcutaneous and high intraperitoneal tumorigenicity.

It must also be remembered that the majority of cell lines were established from highly advanced tumors. Their molecular profile reflects the sum of genetic changes accumulating during consecutive cycles of chemotherapy and recurrences, as well as accumulated changes necessary to adapt to the in vitro culture conditions [43,55].

##### Patient Derived Xenografts (PDXs)

Patient-derived xenografts (PDXs) are produced by direct engraftment of clinical samples into immunodeficient mice (reviewed in the work of [45]). Not only surgically resected tumors, but also samples from ascites can be used. After some time, usually 2−4 months, PDXs attain logarithmic growth phase and the tumor can be harvested for analysis and/or for serial transplantation. The take rate, which varies in different reports between 25% and 95%, and tumor latency depend on the properties of the tumor and mice strain. PDX models are of particular value if they are accompanied by detailed clinical information and are well characterized molecularly (in respect to gene mutations, copy number variations, gene expression pattern, and so on).

PDXs contain stromal and vascular elements of original tumor, as well as human cellular elements of immunological system and stem cells; however, it is not clear how long they last. Comparison of gene expression profile of paired PDX and donor tumors from nine ovarian cancer patients revealed almost 2000 differentially expressed genes. Over 90% of these genes were down-regulated in PDXs and enriched in stroma-specific functions. It is speculated that these differences are predominately because of the loss of human stroma in PDX tissues. Other differences in gene expression may reflect changes required for a human tumor to adapt to a murine host [56,57]. It was also observed that several copy number alterations, characteristic for primary tumors of different origin, gradually disappear in PDXs, probably owing to the loss of selective pressure during propagation in mice [58]. Nevertheless, it is accepted that early-stage PDXs are suitable for studies on epithelial–stromal interactions and on the biology of cancer stem-like cells.

PDXs are particularly well suited for drug discovery and preclinical studies on new therapies, as they relatively closely resemble human cancers [53]. A high correlation between patient drug response and PDX response to the same drug was observed in PDX models derived from ovarian cancer patients in several studies, (e.g., the works of [59,60]). The PDX model was also used to test HER2-targeted therapy. Three out of the tested PDXs were identified by new generation sequencing to have alterations in genes encoding members of the ERBB2 pathway. In these PDXs, when chemotherapy and HER2-targeted therapy (pertuzumab/trastuzumab) were administered together, a significant regression of tumor was observed after six weeks of treatment, compared with chemotherapy alone [61].

PDXs also have their limitations; the main disadvantage is a lack of host immunological response in immunocompromised mice. Xenografted mice are not suited for studies on cancer immunotherapy and host–cancer cell interactions [41]. To overcome these issues, attempts are made to develop humanized PDX recipient mice, for example, by intravenous injection of CD34+ cells from the same patient (reviewed in the work of [62]).

Other disadvantages of the PDX model are its price (immunocompromised mice are expensive to purchase and to maintain), special requirements concerning facilities needed to accommodate these rodents, technical complications concerned with tumor transplantation, and a lack of stability (clonal selection in consecutive passages and progressive loss of human stromal component).

#### 2.2.2. Syngeneic Model

In a syngeneic model, cancer cells are derived from the same mouse strain and are introduced into the immunocompetent host. It is, however, an entirely rodent model (mouse cancer cells propagated in mouse organism), thus the results may not translate into the situation in humans. The advantage offered by this model is the opportunity to study anticancer immune response, epithelial–stromal interactions, and tumor vascularization. In addition, the risk of infection is lower than in immunocompromised mice.

Classical syngeneic model is based on ID8 cells (spontaneously transformed OSE from C57BL/6 mice), injected into mice of maternal strain. The cells are able to form intraperitoneal metastases after intrabursal injection. The model is useful for studies on tumor growth and metastasis on the background of functional immunological system [63].

A similar model was obtained for FVB/N mice. After several years of establishing and growing cultures of FVB/N mouse OSE, one cell line underwent spontaneous neoplastic transformation. After intrabursal inoculation to the syngeneic host, these cells eventually gave multiple tumors disseminated within peritoneal cavity, accompanied by formation of ascites. It was shown that a subpopulation of these cells expressing stem cell antigen 1 (Sca1) appeared to be more tumorigenic than the SCA1(−) population. The authors postulated that the obtained model corresponded to the human HGSOC. In our opinion, it is not certain, as the tumors’ histology was mixed, including regions of mucinous, undifferentiated, and papillary serous structures. Although tumors were positive for Müllerian marker Pax8, they had no p53 mutation, which is typical for HGSOC [64]. Nevertheless, the model offers vast potential for the testing of novel therapeutics, including immune therapies. It will also allow for the discovery of new screening targets that can be involved in the malignant transformation of OSE.

#### 2.2.3. Genetically Modified Mice

Genetically modified mice models are widely used to study the role of certain oncogenes and suppressor genes in the initiation and progression of many cancers. However, in the case of ovarian cancer, infertility that develops in the course of the disease is a major limitation in obtaining such a model by classical transgenesis. Wider application of transgenic models has become possible since the invention of newer techniques like the Cre/Lox system, which enable conditional knock-in and knock-out of selected genes. The Cre/Lox system can be combined, for example, with the tetracycline-controlled transcriptional activation system, in order to achieve genetic modification at a specific time point. In order to obtain tissue- or organ-specific modification, selected promoters are used, although there are some discrepancies concerning their exact tissue-specificity. Alternatively, the constructs can be applied intrabursally, which is thought to result in specific modification of OSE. However, this technique is also not absolutely precise. Two methods of injecting adenovirus into the bursal cavity were compared (LacZ activity, resulting in blue staining, was present in the modified cells)—first through the uterine–tubal junction and the oviduct, and second through the infundibulum [65]. Both methods allowed for effective modification of OSE cells; the second was more tissue-restrictive, but traces of blue staining could also be detected in the oviduct and in the bursa. Such technical constraints impede definite identification of the cell-of-origin of the tumors developing as a result of genetic modifications introduced by these means.

Below, we will highlight some sophisticated model systems that were used to identify genes crucial for ovarian cancer initiation and to investigate its tissue(s) of origin. Some of these models were also used for drug testing and the evaluation of molecular mechanisms of drug-response [66].

Sandra Orsulic et al. performed a large cycle of studies based on the combined use of genetically modified mice, in vitro genetic modifications, and the syngeneic model. These studies were focused on the mechanism of neoplastic transformation in OSE.

**Figure 2:**
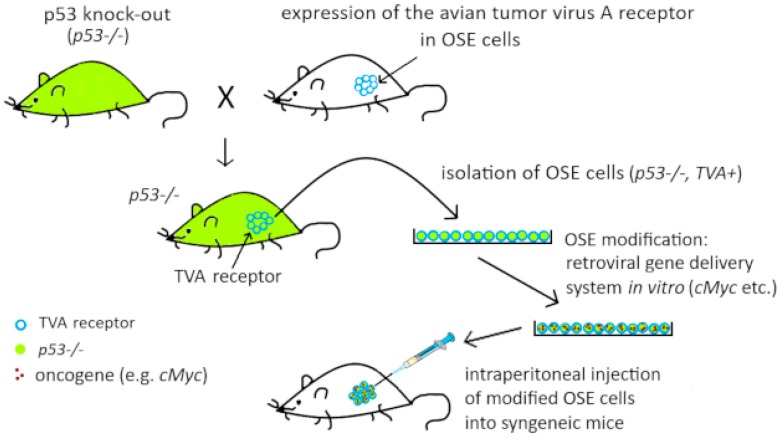
Mouse model designed by Orsulic et al. [67] to evaluate oncogenes necessary for neoplastic transformation of ovarian surface epithelium (OSE) cells with an inactive *p53* gene. TVA, tumor virus receptor A.

The first model was constructed to examine which oncogenes are indispensable for the transformation of OSE cells having inactive *p53* (Figure 2). The *p53* knock-out animals were crossed with mice bearing the transgene containing avian tumor virus receptor A (TVA), under control of specific primer(s). The resulting offspring were characterized by a lack of p53 protein in all tissues and, additionally, they demonstrated membrane expression of TVA in OSE cells. The ovaries were explanted and further modifications were carried out in vitro, using the retroviral transfer system, taking advantage of the TVA receptor. Thus, different oncogenes could be introduced into *p53−/−* cells. These cells were further injected, either subcutaneously or intrabursally, into the syngeneic mice and their tumorigenicity was assessed.

It was found that for neoplastic transformation, OSE cells with dysfunctional *p53* require any two of these three oncogenes: *cMyc*, *K-ras*, and *Akt*. Eight weeks after inoculation with such cells, mice had developed poorly differentiated tumors resembling HGSOC. Subsequent experiments showed that the same modifications did not lead to transformation of other ovarian cells, like granulosa cells and stromal cells. Similarly, OSE cells that had functional *p53* did not undergo transformation, despite oncogenes’ activation.

A similar experimental system was used to test tumor cells’ sensitivity to rapamycin, the mTOR inhibitor. It was shown that rapamycin was efficient in some tumors with active *Akt*; other tumors were resistant, despite having functional *Akt*. The development of latter tumors could be suppressed by combination of rapamycine and MEK inhibitor—PD98059 [68]. This shows that ovarian cancer cells can utilize several signaling pathways to promote proliferation. Each pathway must be blocked to achieve tumor suppression.

Orsulic and coworkers also tested mechanisms of carcinogenesis in the cells depleted of functional *Brca1* and *p53* genes. Triple transgenic mice were used, with floxed *p53* and *Brca1* genes and with membrane expression of the TVA receptor. Ex vivo fragments of the ovary were modified via transduction with viral construct containing Cre recombinase coding sequences. This caused excision of *p53* and *Brca1*. Another viral vector was then applied, containing one of the following oncogenes: *K-ras*, *HER-2*, *Akt*, or *cMyc*. OSE cells modified in that way were injected intraperitoneally into syngeneic mice and tumor development was observed. It was shown that *cMyc* overexpression in OSE cells with *Brca1* and *p53* knock-out leads to neoplastic transformation. Arising tumors showed papillary histology and spread intraperitoneally with the formation of malignant ascites. Except for *cMyc*, none of the other tested oncogenes—*K-ras*, *HER-2*, and *Akt*—could induce neoplastic transformation of the *p53−/−;Brca1−/−* cells. Similarly, *cMyc* overexpression in OSE cells with single knock-out, either in *Brca1* or *p53 locus,* was insufficient to cause transformation [69].

**Figure 3:**
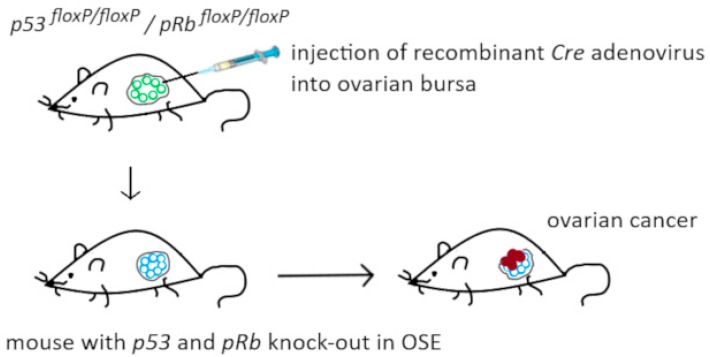
Mouse model used by Flesken-Nikitin et al. [71] for specific knock-out of *p53* and *pRb* genes in OSE cells.

Andrea Flesken-Nikitin et al. constructed a mice model with local knock-out of *p53* and *pRb* in the ovary. They used mice with floxed *p53* and *pRb* alleles and injected intrabursally the construct coding for Cre recombinase. This resulted in excision of both genes in OSE cells. After long-term observation, 33 out of 34 mice with excised *p53* and *pRb* developed malignant ovarian neoplasms (Figure 3). In some mice, formation of ascites was observed, as well as metastases into the lungs, the liver and the second unaffected ovary. However, tumor development was slow: *p53* and *pRb* knock-out was performed on day 60, while the median endpoint was 227 days. Single knock-out of *p53* resulted only in the formation of benign tumors that developed in merely 2 out of 31 mice [70].

Recently, Fleskin-Nikitin et al. discovered the niche of OSE stem cells that is located in the hilum region of mice ovary, at the junction between three types of epithelia—OSE, fallopian tube epithelium (FTE), and mesothelium. These stem cells undergo transformation after *p53* and *pRb* knock-out significantly faster and more efficiently than differentiated OSE cells [71].

Several studies have also shown that malignant transformation and formation of apparent ovarian cancers can be initiated in FTE. In these experiments, tissue-specific genetic modification was achieved using promoters that are specifically activated in Müllerian epithelia. Kim et al. deleted floxed *Pten* and *Dicer1* alleles using Cre recombinase under control of MISRII/Amhr2 (Müllerian inhibitory substance type 2 receptor/anti-Müllerian hormone type 2 receptor) promoter. As a result, they obtained highly malignant neoplasms in the mice fallopian tube fimbriae. Neoplastic foci quickly spread to the ovaries and phenotypically resembled HGSOC [72]. However, as noted by others [73], this model failed to recapitulate the early events in human HGSOC pathogenesis, that is, formation of serous tubal intraepitelial carcinomas (STIC).

**Figure 4:**
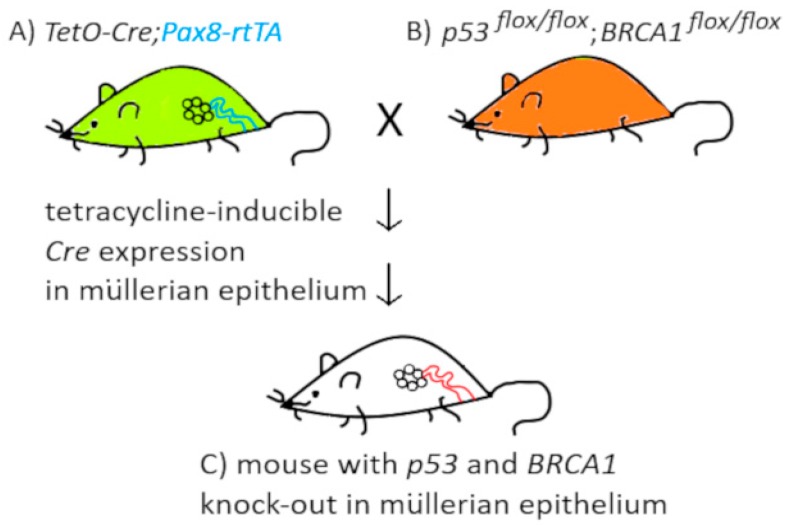
Mouse model used by Perets et al. [73] for the tissue-specific knock-out of the *p53* and *Brca1* genes, using Cre recombinase, the expression of which was limited to the Müllerian epithelia and induced after administration of the tetracycline.

Tissue-specific, tetracycline induced inactivation of the genes of interest was achieved using mice with Cre recombinase under the control of tetracycline promoter, crossed with mice having tetracycline transactivator controlled by PAX8 promoter, specific for Müllerian epithelia. In the offspring, Cre recombinase is induced in Müllerian tissues after administration of tetracycline, while it is absent in OSE (Figure 4). Next, crosses allowed for obtaining animals with specific inactivation of *Brca1* or *Brca2*, and *p53* and/or *Pten*.

Using the above model, it was shown that *Brca2−/−;p53−/−* mice could develop tumors, but after longer latency than *Brca1−/−;p53−/−;Pten−/−* mice and *Brca2−/−;p53−/−;Pten−/−* mice. These experiments showed that loss of function of *Brca1/2* and *p53* together with *Pten* results in serous tubal intra-epithelial carcinomas (STIC) development and its metastases to the ovary and peritoneum. Tumor histology, the pattern of metastasis, and the gene expression profile were very similar to the human HGSOC [73].

The above described models allowed for the activation or inactivation of the given gene(s) in the selected tissues and at the chosen time point. Thus, it was possible to obtain a viable mice strain and induce tumors in selected animals. It is much more difficult to achieve this using standard transgenic animals. One of the very few models obtained by classical transgenesis used oncogenic SV40 sequences (large and small T antigens), under the control of the *MISRII/*Amhr2** promoter. This promoter was shown by the authors to exhibit efficient tissue specific expression in OSE and much weaker expression in the fallopian tube. Before reaching 13 weeks, half of the transgenic mice had aggressive, poorly differentiated tumors that metastasized within peritoneum. None of the females were fertile; the stable strain was generated using a TgMISRII-Tag-DR26 male. In the consecutive generation, all females developed bilateral ovarian cancer resembling human HGSOC [74]. According to the authors of [66], tumors developing in these mice resembled most closely the human low-grade invasive ovarian cancer, but they also had poorly differentiated regions. In our opinion, owing to limited specificity of the promoter used, these cancers could originate both from the OSE and from FTE.

This model has been used to test the chemo-preventive efficacy of RAD001 (Everolimus), an mTOR inhibitor. TgMISRII-Tag mice, which were treated with RAD001 between the 5th and 20th week, showed delayed onset and progression of tumors compared with the placebo group. More tumors in the treated group were contained in the uninterrupted capsule, and less animals had intraperitoneal metastases and ascites [75].

Another transgenic model was obtained using SV40 large T antigen under the control of OGP (oviductal glycoprotein) promoter. Tumors developed in Müllerian structures; namely, the fallopian tubes, endometrium, myometrium, and vagina [76].

It was also shown in the mice model that endometrioid cancers can originate from OSE cells. Activation of oncogenic *Kras* allele in OSE, achieved by intrabursal injection of recombinant adenoviral vector expressing Cre recombinase, resulted in benign epithelial lesions that were classified as endometriosis-like. Similar lesions developed after conditional knock-out of *Pten* in OSE cells. In addition, 7 out of 15 mice with activated *Kras* developed peritoneal endometriosis. The authors suppose that the ovarian and peritoneal lesions may have distinct origins, with the ovarian lesions arising from the OSE and the peritoneal lesions having a uterine or tubal origin. Simultaneous *Pten* deletion and *Kras* activation resulted in the induction of invasive and widely metastatic endometrioid ovarian adenocarcinomas with complete penetrance and a disease latency of only seven weeks [65].

Interestingly, in another study, *Kras* induction and *Pten* deletion resulted in low-grade ovarian serous papillary adenocarcinomas. In this model, genetic modifications were introduced into OSE cells by Cre recombinase expressed under control of the *MISRII/Amhr2* promoter. The tumors developed at an early age and with 100% penetrance [77].

Other authors observed very aggressive tumors, morphologically similar to human endometrioid adenocarcinomas, developing in mice with deregulated Wnt/β-Catenin and PI3K/Pten pathways. Conditional inactivation of the *Pten* and *Apc* genes was obtained by intrabursal injection of Ad–Cre construct into mice with floxed *Apc* and *Pten* alleles. Tumors were already observed six weeks after injection, with 100% penetrance and rapid progression to metastatic disease in up to 75% of animals [78]. The gene knock-out was believed to occur in OSE cells, but the previously mentioned experiments performed by the authors of [65] showed that Cre expression in other (e.g., fallopian) tissues could not be excluded after intrabursal injection of Ad–Cre construct.

In summary, thanks to the novel technologies allowing conditional knock-in and knock-out of selected genes in mice tissues, many sophisticated models were generated, which allow studying the etiology of ovarian cancer. These models helped to elucidate the role of several oncogenes and tumor suppressor genes and their engagement in different histological types of ovarian cancer (Table 3).

Many studies suggest that what we call ovarian cancer can arise either from OSE or from FTE. Current methods of conditional modification of gene expression are still imperfect. The promoters regarded as tissue-specific are in fact not definitely characterized according to their target cells/tissues (widely discussed in the work of [90]). Similarly, leakage may occur after intrabursal injection of viral constructs. Thus, further investigations are necessary to clarify the cellular origin of tumors and to distinguish tissue-specific malignancy from the more general oncogenic properties of the genes analyzed in the described transgenic models.

### 2.3. Xenopus Model

*Xenopus* (clawed frog) is a genus of African frogs, of which two species, *X. laevis* and *X. tropicalis*, are widely used as model organisms. Female *Xenopus* is highly sensitive to human chorionic gonadotropin (hCG), which stimulates their oogenesis. Therefore, they were formerly used as a natural pregnancy test [91]. *Xenopus* models have been used primarily for studies on cell and developmental biology, but they contributed to cancer research by exploitation of the pararell between the mechanisms of embryogenesis and tumorigenesis. In particular, *Xenopus* models are useful to study evolutionarily conserved mechanisms of the cell cycle regulation, cellular signaling cascades, EMT, and cellular metabolism [92,93].

Spontaneous tumors in *Xenopus* are rare; however, ovarian germ cell tumors (dysgerminoma) have been observed in 5–7-year-old frogs after long-term hCG treatment administered for breeding purposes. The relative resistance of *Xenopus* to spontaneous and transplanted tumors has provided an intriguing model to study the mechanisms of tumor immunity. These frogs present a variety of anti-tumoral immune effectors including conventional CTLs, classical MHC class Ia unrestricted CTLs, CD8 NKT-like cells, and NK cells. It is postulated that genetically modified *Xenopus* models that are now under rapid development, could provide an good alternative to the murine system for studying tumorigenesis and tumor immunity [6]. It is also stressed that *Xenopus* models can be generated at a shorter time-scales and at considerably smaller expense than mouse models [92].

### 2.4. Laying Hen Model

The laying hen (*Gallus domesticus*) is the only animal model that spontaneously develops ovarian cancer (reviewed in the literature [7,90,94]). Despite anatomical and physiological differences (see Table 1), the etiology and pathogenesis of ovarian cancer are similar to those in humans. Ovarian cancer in the hen is highly malignant and follows a pattern of dissemination, metastasis, and formation of ascites similar to the human disease. Four histological types of ovarian cancer are found in hen: serous, endometrioid, mucinous, and clear cell, in addition to tumors of mixed histology [95].

Similar to human females, the risk of ovarian cancer development in the hen is highly correlated with age and number of ovulations. In hens, the ovulatory cycle lasts 24–26 h. It is estimated that a two-year-old hen has completed a number of ovulations similar to the average number of ovulations in a woman at menopause. Ovarian cancer occurrence in laying hens ranges from 5% to 35%, depending on the genetic strain and other factors. As in humans, the follicular development and ovulation cycles of hens are under the control of the pituitary gonadotropins and steroid hormones produced by the ovaries. Progesterone (progestin) administration was shown to reduce the ovarian cancer risk in hens by 15% to 90%, depending on the trial [reviewed in the work of [94]]. Risk reduction was accompanied by a decreased number of ovulations. These results suggest that progesterone may have a protective function, which corresponds to epidemiological observations in humans, where prolonged periods with an increased level of progesterone (pregnancy, hormonal contraception) impact on reducing the risk of ovarian cancer.

The described similarities, as well as the relatively rapid development of tumors, the ease of manipulation of external factors (nutrition, hormones, and drug administration), and the availability of strains with different genetic profiles, make the laying hen an important model for research on the biology and treatment of ovarian cancer, as well as for chemoprevention trials.

The similarity between hen and human ovarian cancer is also observed at the molecular level. CA-125, as well as epithelial markers occurring in human ovarian cancer, such as cytokeratin, EGFR, HER-2/neu, VEGF, COX-1, CYP1B1, E-cadherin, and PCNA, appear also in the hen model (reviewed in the work of [96]). *p53* mutations are observed in about 50% of hen ovarian cancer cases. As in women, *Ras* mutations in hens are less common.

Controversies about the cellular origin of ovarian cancer concern both humans and hens. In hen, similar to human females, both the ovary and the oviduct are often involved at the time of cancer diagnosis. In laying hens, expression of oviduct related proteins, such as OVA (ovoalbumin), OVOS2 (ovostatin 2), PAX2, or EGFR1, was frequently reported in ovarian cancer. A gene expression profiling experiment also revealed expression of several oviduct-related genes in normal oviduct, as well as in early and late stage ovarian tumors, but not in normal ovarian surface epithelium. In fact, 10 of the top 25 up-regulated genes in hen ovarian tumors were oviduct-related [97]. These data suggest that chicken ovarian tumors may arise from alternative sites, including the oviduct. It is possible, however, that their expression is related to transdifferentiation of OSE.

Increased HER-2/neu expression is suggested to be associated with progression of primary fallopian tube carcinoma and HGSC in humans. Overexpression of HER-2/neu is also observed in some ovarian carcinomas in hens; this phenomenon is discussed in the context of fallopian origin of these cancers [90].

#### Chorioallantoic Membrane Model

The chicken egg chorioallantoic membrane (CAM) is a highly vascularized extra-embryonic structure, which can be conveniently used for tumor propagation. The model is relatively simple, rapid, and cost-effective. The egg can be incubated in ovo or ex ovo. The latter model has the advantage of better visibility and greater ease of experimental manipulation, such as tumor cells’ inoculation and intravenous injection of analyzed compounds, but the embryos have a lower survival rate. The highly vascularized structure of CAM and nutrient-rich environment allow for rapid tumor formation, usually within 3–5 days after inoculation with cancer cells. Tumors obtained from established cancer cell lines develop abundant vasculature and elements of extracellular matrix; thus, cancer cells grow in more natural microenvironments. CAM assays have already been widely used to study angiogenesis, tumor cell invasion, and metastasis, but this model also allows for simultaneous screening of large numbers of pharmacologic agents, and is an alternative to the in vivo PDX model (reviewed in the literature [8,98,99]).

The chorioallantoic membrane (CAM) develops from the seventh day of incubation. The recommended time for inoculation is day 9. The embryo is not fully immunocompetent until the 18th day. However, nonspecific inflammatory response may occur after 15 days of development. It is less likely to occur when the test material is grafted as soon as the CAM begins to develop, while the host immune system is immature. The recommended time for harvest is the 16th day of incubation (seven days after inoculation). Alternatively, tumors may be harvested the day before tumor rejection would likely occur at day 18 (reviewed in the work of [98]). A certain limitation is imposed by the rapid development of chickens, as the time from CAM formulation to chicken hatching is only two weeks [100]. The CAM model does not require ethics committee approval for animal experiments, because in most countries, the chick embryo is not considered a live animal until day 17 of development. The CAM is not innervated, and experiments are terminated before the development of centers in the brain associated with pain perception (reviewed in the work of [8]).

The CAM model of ovarian cancer was used to evaluate the efficacy of anticancer drug delivery by means of the recently developed biodegradable nanoparticulate carrier, PMO (periodic mesoporous organosilica). It was shown that nanoparticle formulation enabled a higher amount of doxorubicin to be administered safely and preferentially to the tumor, and enabled effective tumor elimination. Two million OVCAR-8/GFP cells were transplanted by day 10 onto the CAM. By day 13, tumors that closely resembled the human ovarian cancer were formed. Then, the PMOs loaded with doxorubicin were administered intravenously. All chicken survived when injected with nanoparticles containing up to 200 μg of doxorubicin and internal organs appeared normal. In the case of free doxorubicin, none of the eggs survived 200 μg of doxorubicin, while 100 μg of doxorubicin caused significant damage to various organs in the embryo. Next, the PDXs of HGSOC were propagated on the CAM. After PMO–doxorubicin injection, these tumors were eliminated within three days [101].

CAM assay was also used to monitor the metastatic properties of ovarian cancer cell lines OVCAR-3, SKOV-3, and OV-90, as well as to study the effect of potential therapeutic molecules [102]. Ovarian cancer cells (9 × 10^5^ cells) were mixed with Matrigel and grafted on top of the CAM at day 11. The invasion of the ovarian cancer cells through the ectoderm into the mesoderm was assessed on day 14. The results were consistent with cell motility and invasion observed in in vitro assays. It was also shown that neutralizing antibody against protein A effectively inhibited OV-90 cells invasion into the mesoderm of the CAM, compared with the control anti-mouse IgG, where OV-90 cancer cells invaded the mesoderm and a destruction of the ectoderm was observed. The authors postulate that the described model closely mimics human ovarian cancer metastasis to the peritoneum.

## 3. Conclusions

Animal models are indispensable for both basic and preclinical research. They allow for the better understanding of the etiology and pathogenesis of diseases, identify potential therapeutic targets, and test novel therapeutic approaches. Unfortunately, there is no ideal in vivo system that would allow the modelling of all stages of development and progression of human ovarian cancer and to test drug responses (Table 4). The fruit fly is evolutionarily too far from humans; in mice, ovarian cancer does not develop spontaneously; whereas the hen is an oviparous animal and thus has a distinct physiology and anatomy. Despite these constraints, each of these animals can be used for a particular type of investigation.

Drosophila melanogaster is suitable for basic research. Border cells present in the ovary of fruit fly are an excellent model for the study of cellular migration, invasion, dynamics of intercellular connections, and regulation of epithelial-to-mesenchymal transition. Studies using this model allowed the examination of the functions of Jak/Stat and Hippo signaling pathways, which are responsible for the regulation of migration processes. Additionally, research on the Drosophila model has shown that VEGF and EGFR factors are involved in the regulation of cell migration. Further important studies on the fruit fly model concerned the role of BRCA2 protein in homologous recombination during meiosis and mitosis.

Using evolutionarily higher animals, it is possible to model tumors with their (almost) natural architecture and microenvironment. These models produce peritoneal metastasis and the ascitic fluid, which corresponds to the clinical situation in humans and allows for the study of these processes.

In a syngeneic model, mice are implanted with tumor cells derived from animals of the same strain. This model allows for the study of tumor architecture, its growth, and metastasis, as well as the anti-tumor response in an immunocompetent host.

The xenograft model allows the study of tumors arising from human ovarian cancer cells that develop in immunodeficient mice. This model is useful mainly for preclinical studies, but excluding immunotherapy. Current emerging immunotherapies have shown promising results in other cancer types and should be exploited in ovarian cancer. The major barrier in immunotherapy for ovarian cancer is considered to be the highly immunosuppressive tumor microenvironment. It is suggested that *Xenopus* models could be developed and adapted for the studies of the cross-talk between the tumor and immune system [6,103].

Wider applicability of the genetically modified mouse models emerged with the development of methods allowing for conditional knock-in and knock-out of selected genes at a given time point and in a specific organ or tissue. These models enriched our knowledge about the tissue origin of ovarian cancers, genetic events necessary for tumor initiation and determining its malignancy. Using genetically modified mice, it was shown that high-grade serous ovarian cancer can be initiated in both the ovarian surface epithelium and the fallopian tube epithelium. However, in our opinion, the question about the tissue/cell of origin of each histological type of ovarian cancer has not been definitely resolved, so far.

The advantage of the laying hen model is that ovarian cancer develops spontaneously in these birds. This provides the opportunity to study genetic, biochemical, and environmental risk factors, as well as the initiation and progression of cancer and its histological origin; the model can also be used for drug testing. The chicken egg model is also very attractive, with a human tumor developing in the chorioallantoic membrane. The model is simple, rapid, and cost-effective; it is particularly well suited for drug-response studies.

## Figures and Tables

**Table 1:** Similarities and differences between animal models and human ovarian cancer (partially based on the work of [9]).

Features		Human	Fruit Fly	Clawed Frog	Mouse	Laying Hen
**Anatomic**	Ovaries (number)	+ (2)	+ (2)	+ (2)	+ (2)	+ (1)
	Fallopian tubes (number)	+ (2)	+ (1)	+ (2)	+ (2)	+ (1)
	Fallopian tube fimbriae	+	−	−	+	−
	Ovarian bursa	−	−	−	+	−
	Uterus	+	+	− ^1^	+	+ ^2^
Histological	Endometriosis	+	−	−	− ^3^	−
Physiological	Menstrual cycle	+	−	−	−	−
	Estrus cycle	−	−	−	+	−
Clinical	Spontaneous cancer development	+	−	+	−	+
Pathological	Confirmed histotypes	+	−	−	+	− ^4^
Experimental	Genetic modifications	NA	+	+	+	−

^1^ In frogs, the fallopian tube disembogues into cloaca. ^2^ The hen uterus lacks endometrium; it is responsible for egg shell formation. ^3^ Endometriosis in mice does not develop spontaneously; its models are obtained surgically or by genetic engineering. ^4^ Histological types of hen tumors were not tested for immunophenotypic similarity to human ovarian cancers. NA—not applicable.

**Table 2:** Features of xenograft models depending on the site of cancer cells administration.

Injection Site	Features of the Model
Subcutaneously	Tumor is limited to the site of cells’ injection Easy observation of tumor growth Tumor develops in an unusual anatomical location and microenvironment Not feasible to study angiogenesis No metastases and disease progression are observed
Intraperitoneally	A good model of disseminated disease Metastases to the peritoneum and diaphragm with ascites formation Tumor growth can be monitored using in vivo fluorescence or luminescence techniques Not suitable for investigating the initiation of the neoplastic process and the early stages of disease
Orthotopically	Tumor develops in a closed space limited by the ovarian bursa Good model for research on early stages of disease Metastasis and progression of disease not always occur in this model Technically difficult (leakage of cancer cells outside bursa may occur)

**Table 3:** Genetically modified mouse models for ovarian cancer research.

Genotype(s)	Postulated Tissue of Tumor Origin	Histology of The Tumors	References
*(p53−/−;cMyc+;Kras+)* *(p53−/−;Kras+;Akt+)* *(p53−/−;Akt+;cMyc+)* *(p53−/−;Akt+;cMyc+;Kras+)*	OSE	primary tumors: poorly differentiated carcinomas; metastasis: poorly differentiated carcinomas and papillary serous carcinomas	Orsulic et al. (2002) [67]
*(p53−/−;pRb−/−)*	OSE	poorly differentiated carcinomas;	Connolly et al. (2003) [74]
*(Hoxa9+)* *(Hoxa10+)* *(Hoxa11+)*	OSE	*Hoxa9+*: papillary serous carcinomas; *Hoxa10+*: endometrioid carcinomas; *Hoxa11+*: mucinous carcinomas	Cheng et al. (2005) [79]
*(Kras+;Pten−/−)*	OSE	endometrioid carcinomas	Dinulescu et al. (2005) [65]
*(Brca1−/−;p53−/−;cMyc+)*	OSE	papillary serous carcinomas	Xing and Orsulic (2006) [69]
*(Apc−/−;Pten −/−)*	OSE	endometrioid carcinomas	Wu et al. (2007) [78]
*(p53−/−)* *(Brca1−/−;p53−/−)* *(pRb−/−;p53−/−)* *(pRb−/−;p53−/−;Brca1−/−)*	OSE	malignant leiomyosarcomas	Clark-Knowles et al. (2009) [80]
*(K-ras−/−;Pten−)*	OSE	low-grade serous carcinomas (LGSC)	Fan et al. (2009) [81]
*(Brca1−/−;p53−/−)*	OSE	high-grade leiomyosarcomas	Quinn et al. (2009) [82]
*(Dicer−/−;Pten −/−)*	FTE	high-grade serous carcinomas (HGSC)	Kim et al. (2012) [72]
*(Brca1−/−;p53 −/−;pRb−/−)* *(Brca2−/−;p53−/−;pRb−/−)*	OSE	serous carcinomas	Szabova et al. (2012) [83]
*(Brca1−/−;p53^mut^;Pten−/−)* *(Brca1+/−;p53^mut^;Pten−/−)* *(Brca2−/−;p53^mut^;Pten−/−)* *(Brca2+/−;p53^mut^;Pten−/−)*	FTE	serous tubal intra-epithelial carcinomas (STIC)	Perets et al. (2013) [73]
*(p53−/−)* *(pRb−/−)* *(p53−/−;pRb−/−)*	OSE	*p53−/−*: poorly differentiated CK8-positive neoplasms; *p53−/−;pRb−/−*: well-differentiated serous epithelial neoplasms, poorly differentiated CK8-positive neoplasms, undifferentiated neoplasms	Flesken-Nikitin et al. (2013) [70]
*(p53−/−;Top2a+)*	FTE	serous tubal intra-epithelial carcinomas (STIC)	Sherman-Baust et al. (2014) [84]
*(Lkb1−/−;Pten−/−)*	OSE	high-grade serous carcinomas (HGSC)	Tanwar et al. (2014) [85]
*(Kras+;Pten−/−)* *(Muc1+;Kras+;Pten−/−)*	OSE, FTE	endometrioid carcinomas	Tirodkar et al. (2014) [86]
*(p53^mut^;Dicer−;Pten−)*	OSE	high-grade serous carcinomas (HGSC)	Kim et al. (2015) [87]
*(p53+/+;Pten−/−;Kras+)* *(p53+/−;Pten−/−;Kras+)* *(p53−/−;Pten−/−;Kras+)*	OSE	*p53+/+;Pten−/−;Kras+*: low-grade serous carcinomas (LGSC); *p53+/−;Pten−/−;Kras+*: invasive serous carcinomas, mucinous carcinomas; *p53−/−;Pten−/−;Kras+*: likely early serous carcinomas	Ren et al. (2016) [88]
*(Apc−/−;Pten−/−)*	FTE, OSE	OSE: poorly differentiated carcinomas; FTE: well-differentiated tumors	Wu et al. (2016) [89]

FTE—fallopian tube epithelium, OSE—ovarian surface epithelium.

**Table 4:** Advantages and limitations of ovarian cancer models. EMT—epithelial-to-mesenchymal transition.

Ovarian Cancer Models	Advantages	Limitations
Fruit fly(*Drosophila melanogaster*)	Suitable for basic research Simple structure, short life cycle, easy propagation and maintenance Conserved DNA repair mechanisms and signaling pathways Border cells from ovary are suitable for migration, invasion, cellular mobility and EMT studies	Simple anatomy and physiology Simple immune system Tumors require induction and have a poor metastatic potential
Clawed frog(*Xenopus ****laevis, Xenopus tropicallis***)	Good model for studies of cell and developmental biology Conserved signaling pathways High fertility, cost-effective maintenance Can be suitable for studies on tumor immunity and anticancer immune response	Spontaneous tumors are rare Etiology of *Xenopus* ovarian cancer is not known Carcinogens used in mammals do not cause malignant tumors in *X. laevis* Suitable models of epithelial ovarian cancer must be developed
Mouse (*Mus musculus*)		
Xenograft mouse models	Possibility of propagation of human cancers Tumor cells may be derived from a cell culture or patients’ tumor (PDX model) Good model of advanced disease Possibility to study the tumor microenvironment Suitable for drug response testing and validation of new therapies	Time consuming construction of the model High cost of model construction and maintenance of immunodeficient mice No host immune response Not suitable for immunotherapy and host–cancer cells interactions studies
PDX (patient-derived xenografts) mouse models	Retains the original characteristics of the tumor (histology, mutation status, changes in the number of DNA copies, gene expression) Contains elements of human tumor microenvironment (cancer stem cells, microvascularization, memory T cells) High correlation between PDX and patients’ clinical response	Time-consuming construction of the model High cost of model construction and maintenance Limited access to biological material Not suitable for immunotherapy and host–cancer cells interactions studies Human stroma elements are exchanged with time for mouse equivalents
Syngeneic mouse models	Good model for basic research and preclinical studies Immunocompetent host Possibility to test the anti-cancer immune response Possibility to study the tumor microenvironment, its vascularization, and epithelial–stromal interactions Reduced risk of infection in mice	Model based entirely on the animal system
Genetically-engineered mouse models	Good model for basic research and for studies on ovarian cancer initiation and progression Possibility to obtain tissue-specific modifications Ability to study the genetic events necessary for the initiation of carcinogenesis	Necessity of cancer induction Model difficult to design owing to poor understanding the tissue of origin of ovarian cancer Time-consuming and costly construction of the model Deficiency of tissue-specific promoters
Hen (*Gallus domesticus*)		
Laying hen	Spontaneous development of cancer Short time of tumor formation Suitable for studies on genetic, biochemical, and environmental risk factors; initiation and progression of cancer; and its histological origin Different strains and genetic profiles	Anatomical and physiological differences between hens and humans Lower incidence of histological types that are predominant in humans No species-specific antibodies No knock-out models
Chicken chorioallantoic membrane (CAM)	Short time of tumor formation Possibility to study tumors derived from human cancer cell lines and PDX Rich vasculature and nutrient content of CAM Well-developed extracellular matrix of the tumor Suitable to study angiogenesis, tumor development and metastasis, drug-response, and so on Low costs	Short time from CAM formation to chicken hatching
